# Progress and challenges in TB vaccine development

**DOI:** 10.12688/f1000research.13588.1

**Published:** 2018-02-16

**Authors:** Gerald Voss, Danilo Casimiro, Olivier Neyrolles, Ann Williams, Stefan H.E. Kaufmann, Helen McShane, Mark Hatherill, Helen A Fletcher

**Affiliations:** 1 Tuberculosis Vaccine Initiative (TBVI), Lelystad, Netherlands; 2 Aeras Global TB Vaccine Foundation, Rockville, MD, 20850, USA; 3 Sanofi Pasteur, Swiftwater, PA, 18370, USA; 4 Institut de Pharmacologie et de Biologie Structurale, Université de Toulouse, CNRS, UPS, Toulouse, France; 5 Centre for Emergency Preparedness and Response, Public Health England, Salisbury, UK; 6 Max Planck Institute for Infection Biology, Berlin, Germany; 7 The Jenner Institute, Nuffield Department of Clinical Medicine, University of Oxford, Oxford, UK; 8 South African Tuberculosis Vaccine Initiative, Institute of Infectious Disease & Molecular Medicine and Division of Immunology, Department of Pathology, University of Cape Town, Cape Town, South Africa; 9 London School of Hygiene & Tropical Medicine, Immunology & Infection, TB Centre, London, UK

**Keywords:** Tuberculosis, vaccine, BCG, vaccine development, clinical trials

## Abstract

The Bacille Calmette Guerin (BCG) vaccine can provide decades of protection against tuberculosis (TB) disease, and although imperfect, BCG is proof that vaccine mediated protection against TB is a possibility. A new TB vaccine is, therefore, an inevitability; the question is how long will it take us to get there? We have made substantial progress in the development of vaccine platforms, in the identification of antigens and of immune correlates of risk of TB disease. We have also standardized animal models to enable head-to-head comparison and selection of candidate TB vaccines for further development.  To extend our understanding of the safety and immunogenicity of TB vaccines we have performed experimental medicine studies to explore route of administration and have begun to develop controlled human infection models. Driven by a desire to reduce the length and cost of human efficacy trials we have applied novel approaches to later stage clinical development, exploring alternative clinical endpoints to prevention of disease outcomes. Here, global leaders in TB vaccine development discuss the progress made and the challenges that remain. What emerges is that, despite scientific progress, few vaccine candidates have entered clinical trials in the last 5 years and few vaccines in clinical trials have progressed to efficacy trials. Crucially, we have undervalued the knowledge gained from our “failed” trials and fostered a culture of risk aversion that has limited new funding for clinical TB vaccine development. The unintended consequence of this abundance of caution is lack of diversity of new TB vaccine candidates and stagnation of the clinical pipeline. We have a variety of new vaccine platform technologies, mycobacterial antigens and animal and human models.  However, we will not encourage progression of vaccine candidates into clinical trials unless we evaluate and embrace risk in pursuit of vaccine development.

## Introduction

Tuberculosis (TB) is the leading infectious cause of death world-wide (WHO TB report 2017). Current measures used for TB control are effective but insufficient. The decline in global TB rates remains incremental and the propensity of *Mycobacterium tuberculosis* (MTB) to develop drug resistance is a serious threat to our ability to control this disease with the currently available tools. Vaccination can be an effective strategy for TB control and it is estimated that Bacille Calmette Guerin (BCG) prevents 120,000 childhood deaths each year ^1^. A TB vaccine that could enhance protection in infancy, or extend protection into adulthood, would have a significant impact on global TB rates ^2, 3^. 

In 2012 the TB vaccine community, led by the TuBerculosis Vaccine Initiative (TBVI) and Aeras, published a Blueprint for TB Vaccine Development: a global, integrated strategy, outlining major scientific challenges, critical activities and crucial questions ^4^. The Blueprint summarized the current state of TB vaccine development and identified key areas of research critical for the development of a new, effective TB vaccine ( Box 1).

Box 1. **TB Vaccine Blueprint, 2012, recommendations**

**1) Technologies and discovery:** A need for better understanding of TB disease, natural resistance, innate, T-cell and antibody responses and diversity in antigen discovery

**2) Preclinical models:** A need for better models to predict efficacy in humans, for standardization, for comparability and a need to publish experimental failure

**3) Biomarkers and immune correlates:** A need to predict vaccine efficacy, for use of new technologies, to understand the role of IFN-γ in protection and for longitudinal studies of correlates of risks

**4) Clinical trials and harmonization:** A need for capacity strengthening of clinical trial sites, to determine appropriate endpoints, to address regulatory and ethical issues and plans for post licensure sustainability.

It was envisioned that the recommendations would guide the next decade of TB vaccine development. This paper summarizes the major advances and achievements since the publication of the Blueprint in 2012 and updates the critical activities and recommendations for accelerating TB vaccine development today.

## TB vaccine technologies

A new and more effective TB vaccine is an inevitability. In TB there is no doubt that immunity can prevent disease and no doubt that protective immunity can be induced by vaccination. Evidence for this includes the long lasting immune protection found following immunisation with BCG ^1, 5, 6^ and natural immunity found in those latently infected with MTB ^7^ and in those who either clear infection or resist disease ^8^. The critical question is when a new TB vaccine will be achieved. The simple answer is that the more we invest in TB vaccine development the sooner we will have an effective TB vaccine.

**Figure 1.**
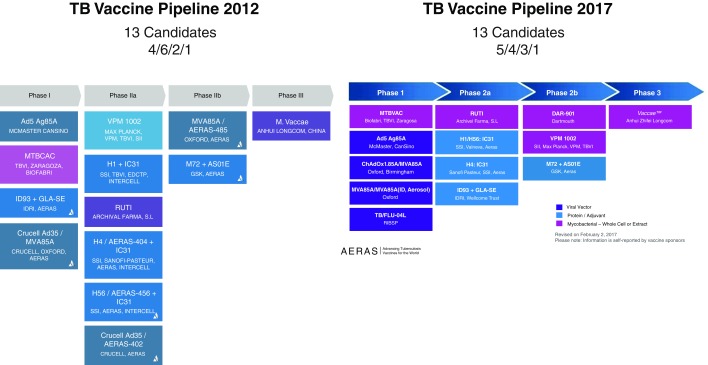
The TB Vaccine Pipeline 2012–2017 (adapted from http://www.aeras.org/pages/global-portfolio).

Reflecting on the TB vaccine pipeline over the last 5 years, we see a small number of candidates that have failed at an early clinical stage, and some new candidates ( Figure 1). Very few pre-clinical candidates have entered the TB vaccine pipeline and those in the pipeline have moved slowly through the stages of vaccine development or have not progressed at all ( Figure 1). A more diverse and dynamic pipeline is needed to accelerate towards our goal of a new TB vaccine. We need to test a broader range of vaccine technologies against a broader range of antigens and we need to move vaccine candidates more rapidly through the pipeline. 

Vaccine technologies that have shown promise in pre-clinical studies include the cytomegalovirus (CMV) vector, which is a live, attenuated, persistent viral vector able to express multiple MTB antigens. It has been shown that the engineering of the CMV vector leads to constant, low-level replication of the virus, giving sustained antigen expression and long-term immunity, making this technology highly attractive for TB vaccine development ^9, 10^. Vaccine technologies under development for the pandemic flu response could also be applied to TB. One such approach uses mRNA as a vaccine vector ^11^. These mRNA vaccines yield high level *in vivo* Ag expression and are relatively simple to manufacture enabling them to be tested against multiple pathogens with relative ease. Antibody inducing vaccines are underrepresented in the current TB vaccine pipeline and although there are many technologies available there is a need for more basic research in this area. It has been shown in a series of non-human primate (NHP) experiments that mucosal or *intravenous* vaccination with whole cell mycobacterial vaccines provides better protection than parenteral vaccination, by inducing more T helper (TH) TH17 cells ^12^, more resident memory T cells ^13^, and more effector T cells ^14^.

The challenge ahead is to increase the rate at which candidate vaccines enter the pipeline and the rate at which candidates move through the pipeline ( Box 2). There are promising new approaches, although obstacles need to be overcome for the use of *intravenous* inoculation or viral vectors which integrate into the host genome. Transparent and robust criteria for moving vaccines from one stage of vaccine development are currently being reviewed and will be used to increase the pace of TB vaccine development.

Box 2. **Conclusions and recommendations**

**TB vaccine technologies**

- The global TB vaccine community should unite to maintain a dynamic vaccine candidate pipeline from discovery to late stage.
- There are currently no unanimously agreed criteria for advancing vaccine candidates.

**TB vaccine research and development**

- Future discovery efforts should include investigation of immune quality and vaccine efficacy in response to combinations of vaccine platform, antigen and adjuvant.
- The evaluation of host factors impacting protection should be included in future scientific investigation.
- Whole cell mycobacterial vaccines should continue to be central to TB vaccine development.

**The role of animal models**

- Animal models and clinical studies should progress in parallel and may offer opportunities for cross-validation.
- The most appropriate animal models should be selected based on evidence and the underlying question(s) to be answered.
- The use of multiple different animal models can have a cumulative value in assessing vaccine candidates or answering pathogenesis questions.
- There is an opportunity for the funders to encourage further standardization of models.
- An obligation to publish animal studies regardless of the outcome (as it is the case for clinical trials) should be encouraged and would facilitate vaccine development.

**Biomarkers, Systems Biology and immune correlates**

- The approach to biomarkers should remain broad, looking at correlates of safety, risk of stable infection or disease, and vaccine efficacy.
- Observational studies will help to identify biomarkers of risk of infection or disease.
- Interventional (vaccine) studies and observational studies should be used to create and expand biobank repositories.

**Experimental medicine and human challenge**

- A space for clinical research studies needs to be maintained and expanded.
- A favourable regulatory environment is critical for the conduct of clinical research studies and should be advocated for.
- Investment into the establishment of controlled human TB challenge models needs to continue. Learnings from the malaria field should be integrated in this process and synergies with biomarker research needs to be created.

**Clinical and late stage development**

- We need to keep (pipeline) diversity at all levels since we still wait for a clear efficacy signal.
- De-risking candidates through gating criteria does not mean being risk-adverse.
- We need to evaluate and accept some risk but prepare carefully and perform high quality studies which can advance the field even in the absence of an efficacy signal.

## TB vaccine research and development

Novel vaccine platform technologies alone will not lead us to a new TB vaccine. A key activity in research and development is the identification of target antigens for insertion into vaccine candidates. In the last five years we have further developed the concept that MTB has distinct phases of growth, which may be associated with active mycobacterial replication, persistence and dormancy ^15^. Antigens associated with active bacterial replication include the early secreted antigens, such as the Ag85 family, ESAT-6 and CFP-10. These antigens have been used extensively in TB vaccine development as they are highly immunogenic and have shown protection in animal models. Antigens in the DosR regulon, however, are associated with dormancy and their use offers the possibility of designing vaccines to more specifically target latent MTB infection (LTBI). Most platform approaches used in vaccine development to date predominantly induce a TH1 cluster of differentiation (CD) CD4+ T cell response. However, in large scale screening experiments antigens that induce a CD8+ T cell response have been identified and can be used with CD8+ T cell inducing vectors to specifically boost a CD8+ T cell response ^16, 17^. There are also platforms and antigens that promote an antibody response or an unconventional T cell response ^10, 18, 19^. The broader range in antigen choice has been matched with the development of novel adjuvants, including synthetic and bio-inspired molecules, which mimic naturally occurring cellular processes for more efficient delivery of vaccine components to the cell. The selected adjuvant can direct the immune response induced and the vaccine developer now has the option of driving immunity towards TH1, TH2, TH17 with the adjuvant selected ^20^.

We now have a greater ability than ever to manipulate the vaccine induced immune response and this can be done at at least three different levels: 1) Choice of vaccine technology; 2) Choice of antigen; 3) Choice of adjuvant. The next question is what type of immune response should be induced?

Our knowledge of protective immunity has greatly increased in the last 5 years and has broadened our awareness of the importance of the interplay between the innate and adaptive immune responses in TB. Correlates of risk studies have identified TypeI/II interferon (IFN) as a risk factor for progression to TB disease in latently infected adolescents ^21^ and bulk T cell activation has been identified as a risk factor for TB in infants and adolescents ^22^. These correlates of risk studies have shown that the underlying host immune environment plays a dominant role in TB disease risk and the impact of this environment on vaccine induced immunity needs to be explored. We also have a greater appreciation that quality rather than quantity of T cells is important for function ^23^. In addition to T cells, it is becoming more apparent that B cells play an important part in immunity to TB ^24^. In particular B cell function is impaired during TB and LTBI, which impacts cellular immunity ^25^. Exploiting antibody-mediated protection against TB, and MTB dissemination in particular, has already shown promise in animal models ^26^.

Despite the emergence of new technologies, whole cell mycobacterial vaccines remain central in TB vaccine development. Complexity of their antigen components including proteins, lipids and glycolipids allows for interaction with innate immune cells and induction of conventional and unconventional T cell responses as well as antibody responses. It has also emerged that BCG itself can manipulate the host immune, environment. BCG can induce epigenetic changes in monocytes, which enhance their capacity for microbial control, not only of mycobacteria but also against unrelated pathogens. This concept of “trained immunity” is thought to result in upregulation of toll-like receptors and CD14 on monocytes ^27^.

How the quality of the immune response is influenced by host environment, route of delivery, vaccine platform, antigen or adjuvant remains largely unexplored. Now that we have the tools to manipulate the vaccine induced immune response, we need to generate data sets exploring how these tools influence immune quality and vaccine efficacy to efficiently select the optimum combinations of vaccine platform, antigen and adjuvant for TB vaccine development ( Box 2).

## The role of animal models

There are several different animal models that are used in the TB vaccine development pipeline and these are useful from the discovery phase right through to advanced pre-clinical development. Animal models are currently perceived as key for demonstration of safety during all stages of development and immunogenicity during early screening, and informative for demonstration of a protective effect against MTB challenge.

Systematic screening in animal models can thus be used to select vaccine candidates that achieve a threshold of safety, immunogenicity and efficacy. There is also the ability to perform comparative head-to-head testing in independent laboratories, for prioritization of the most promising vaccine candidates. Mice, guinea pigs and NHP are the most commonly used species for vaccine testing and study designs vary depending on the animal species, the type of vaccine and the rationale for demonstrating efficacy (e.g. reducing bacterial burden, prolonging survival or preventing reactivation). This complexity in species and study design attempts to reflect complexity of human disease, but it means that there is no single, harmonized animal model that could be used for clear ‘go / no-go’ decisions for candidate TB vaccines. However, there is greater confidence in data that show the efficacy of a candidate in multiple *in vivo* systems, particularly when those studies are conducted in independent laboratories.

There has been considerable progress in animal models since the 2012 blueprint. There is an improved understanding of the strengths and weaknesses of the different models ^28– 30^ and greater recognition of the need for ‘fit-for-purpose’ study designs to achieve robust, quantifiable measures of efficacy. Animal data are now available for many TB vaccine candidates, some of which are undergoing clinical testing. In all cases the degree of protection of the novel vaccines relative to the controls (unvaccinated or BCG), although statistically significant, is not substantial. More clinical efficacy data are needed to know whether this level of protection in animals is predictive of an efficacy signal in humans; this information is needed urgently to provide biological validity of the animal models and to establish whether existing animal models and study designs need to be refined ^28^. There are also efforts to develop models reflective of the more complex environment of target populations. These include infant animal models for neonatal vaccines; post-MTB exposure vaccination; models of co-morbidities, such as diabetes and HIV infection; and models that involve natural transmission of the pathogen.

Data from animal models have become increasingly important to the Stage Gating processes, which aim to assist developers and funders to accelerate candidates from discovery through pre-clinical development. However, negative results are not always reported in the public domain, and therefore the full value of these data to enable lessons to be learnt, has not been realized. Some funders have set requirements for efficacy in NHP to be demonstrated before clinical testing, which highlights the need for stringent thresholds and harmonization in terms of read-outs of vaccine efficacy. An absolute requirement for statistically robust efficacy in NHP is, however, costly and difficult due to limitations of space and animal availability and must be balanced against the cost of collecting data in humans.

## Biomarkers, Systems Biology and immune correlates

Success in studies of individuals with LTBI and active TB patients has led to host biomarkers becoming an integral part of future TB control. Proof of principle has been given that biomarkers can distinguish between active TB disease and LTBI, and evidence is accumulating that biomarkers can predict progression to active TB ^31^. Thus, it has been demonstrated that small-sized biosignatures comprising 3-4 transcripts are capable of reliably discriminating TB disease from LTBI and medium-sized biosignatures comprising 16 transcripts or less allow prediction of active TB by diagnosing incipient, subclinical TB ^21^.

**Figure 2.**
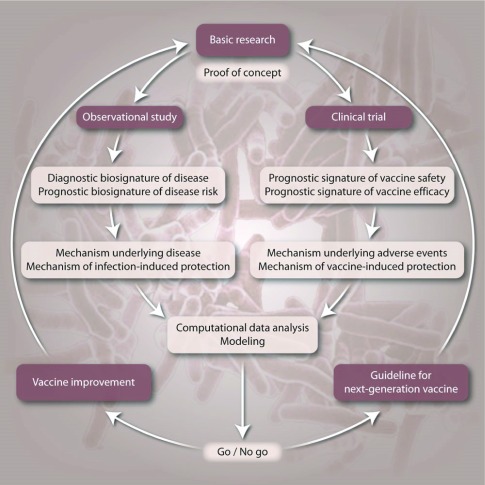
Role of biomarkers in TB vaccine development. This figure has been reproduced with permission from *Kaufmann, Evans, Hanekom, Science Transl. Med., 2015* ^32^.

Beyond their application for diagnosis, biomarkers can provide important contributions to the clinical development of vaccines against TB ( Figure 2) ^32^. First, individuals with subclinical TB may benefit from preventive TB drug treatment. Second, such biosignatures allow stratification of individuals with subclinical TB for clinical vaccine trials to reduce participant numbers and shorten trial duration. Moreover, such biosignatures will serve as valuable tools for monitoring of clinical trial participants. Although they will not replace the clinical endpoints, early recognition of progression to active TB will provide valuable information.

Whilst most biosignatures defined thus far were derived from observational studies on contacts and TB patients, future studies must focus on biosignatures of vaccine efficacy, although these can only be derived when we have a vaccine which demonstrates efficacy in clinical trials. In the meantime, information can be obtained from observational studies on BCG vaccination in infants. It has, for example, been shown that there is a lower risk of progression to TB disease in BCG vaccinated infants with either a higher INF-γ ELISpot response against mycobacterial antigens or higher Ag85A IgG antibody levels ^22^.

Design of biosignatures of vaccine efficacy needs to consider the following groups: individuals who develop active TB despite being vaccinated (vaccine failure), individuals who remain healthy because of vaccination, individuals who remain healthy due to natural resistance (independent of vaccination).

Finally, biosignatures can inform about the mechanisms underlying pathogenesis and protection, paving the way for in-depth analysis of the biological functions of differentially regulated biomarkers. For example, correlates of risk studies, performed using samples collected during the MVA85A vaccine efficacy trial, have revealed that T cell activation and CMV infection are associated with future risk of developing TB disease ^22, 33^. Deeper understanding of the factors that drive TB risk will facilitate the design of next-generation vaccine candidates. 

Integration of biosignatures into clinical trial design will add cost; however, it is critical that we take every opportunity to add value to clinical studies. Biosignatures will be of great value for refining the vaccine candidate tested and for developing alternative vaccine types and modes of immunization.

## Experimental medicine and human challenge

Clinical trials are an essential part of the product development pathway for TB vaccine development. However, restricting the conduct of clinical trials to product development ignores the utility of experimental medicine studies to generate novel scientific insights. Experimental medicine can be defined as ‘an investigation undertaken in humans, relating where appropriate to model systems, to identify mechanisms of pathophysiology or disease, or to demonstrate proof-of-concept evidence of the validity and importance of new discoveries or treatments’. Experimental medicine and product development are not mutually exclusive. A vaccine could be tested to both address a proof-of-concept experimental medicine question and in parallel be a critical step in a product development pathway. An example of this is the first-in-class testing of an attenuated strain of MTB as a potential vaccine candidate ^34^. Furthermore, candidate vaccines could move between experimental medicine and product development, this flexibility is important as we are still at the frontiers of knowledge in TB vaccine clinical testing. There are many examples of small scale, phase I experimental medicine studies which have provided valuable information on safety and immunogenicity, such as the testing of combination vaccine approaches ^35^ and the testing of novel routes of delivery, e.g. aerosol ^36^.

Human challenge models are the ultimate in experimental medicine studies. In such studies, healthy vaccinated volunteers are intentionally inoculated with the pathogen in question, to allow the efficacy of a candidate vaccine to be evaluated in a small-scale study prior to embarking on expensive field efficacy trials. Such controlled human challenge models have been game changing in malaria vaccine development ^37^. However, unlike malaria, and other human challenge models in clinical use, we cannot deliberately infect human subjects with virulent MTB for obvious ethical reasons. Efforts to develop a controlled human mycobacterial challenge model using BCG, or attenuated strains of MTB, are underway ^38, 39^. Ultimately, human challenge models need validation against field efficacy studies. However, they can also be corroborated against a known vaccine effect in preclinical animal models ^38^.

There is an underexploited role for experimental medicine in TB vaccine development, in parallel with product development, in early and late (efficacy) trials. We need innovative ways to demonstrate an efficacy signal in humans with TB vaccine candidates. Controlled human mycobacterial infection studies offer a potential way to achieve this. Such studies, if they were to demonstrate a biological signal, would allow the prioritization of candidates for progression to prevention of disease studies. The predictive value of human immunology, animal models, and these surrogate efficacy endpoints can only be determined by progressing some candidates to field efficacy studies. There is no substitute for human efficacy testing against disease in the development of an effective TB vaccine. Considerable information can be gained from efficacy trials regardless of the efficacy results ^22^. Repeated cycles of iteration between animal and human studies will yield important insights and advance the development of an effective vaccine.

## Clinical and late stage development

Experimental medicine studies are not confined to early phase clinical vaccine trials and human challenge studies, they are also being used to make TB vaccine efficacy trials faster, shorter and more cost-effective. This is achieved by leveraging the much higher incidence rates of MTB infection as measured by interferon-γ release assays (IGRA) conversion for prevention of infection studies (POI) and measurement of TB recurrence after treatment for prevention of recurrence studies (POR). The incidence rates of infection and recurrence are much higher when compared to community-based incident TB disease so clinical trials with POI and POR endpoints can be smaller and faster than those with a disease endpoint (POD). POI and POR trials have rapidly gained acceptance as a pathway to demonstrate proof-of-concept prior to large-scale efficacy trials ^40, 41^. In South Africa, annual IGRA conversion rates of 6–7% in infants ^42^ and up to 14% in adolescents ^43^ have been reported; and TB disease recurrence rates are estimated at 2–5% for standard of care TB treatment arms in a clinical trial setting. These rates are very compelling, in terms of endpoint accrual, compared to the 0.78% annual incidence of TB disease estimated among South African adults by the World Health Organization. As a result, POI trials have been initiated to test the booster vaccines H4 ( NCT02075203) and DAR-901 ( NCT02712424).

The VPM1002 vaccine is being tested in a phase II/III POR trial ( NCT03152903) in adults with cured TB and expects a 10% rate of recurrence (relapse or reinfection) over a 12 month period of follow-up.

Bridging from e.g. a POI trial to POD trial in the same population presents a very real challenge. Using the high TB incidence South African example to illustrate the most cost-effective clinical trial scenario, average incidence of microbiologically confirmed TB disease in adolescents (incidence 0.43%) is almost half that in adults ^44^. Therefore, a POD trial for the identical population in whom POI might be demonstrated as proof of concept would be large, long and costly. Curiously, even though childhood TB is notoriously paucibacillary, it might be more efficient to conduct a POD trial in newborn infants, the only IGRA- population that is not also exposed to other mycobacteria, including BCG, even if the TB disease endpoint were limited to microbiologically confirmed disease (incidence 0.7%) ^42^.

TB vaccine development efforts are increasingly focused on prevention of pulmonary TB disease in adolescents and adults, to block the MTB transmission cycle. The majority of adults in TB endemic countries like South Africa are IGRA+ ^45^, making POD trials in IGRA+ adults more feasible since rates of disease are higher, yet based on our knowledge of historical BCG trials, IGRA+ (and previously BCG vaccinated) adult populations are likely to pose the biggest challenge to demonstrate additional vaccine efficacy ^46^.

Progression of a TB vaccine candidate to POD or a proof-of-concept POI or POR trial is therefore not simply contingent upon application of product development Stage Gate criteria, but a complex consideration of vaccine target population, endpoint accrual, operational efficiency - and most importantly – cost. If we consider the TB vaccine pipeline in 2012 ( Figure 1), which included four vaccines in Phase 1, six in Phase 2, and three in efficacy trials, compared to the five vaccines in Phase 1, four in Phase 2, and four in efficacy trials in 2017 ( Figure 1), the pipeline appears healthy. However, with few exceptions, most candidates have not progressed through the pipeline in the last five years. It is notable that of the seven candidates now in Phase 2, current and planned clinical trial activity includes three POI and three (pilot safety and immunogenicity or efficacy) POR trials, which raises the question of why experimental medicine strategies intended to supplement the traditional product development pathway have instead replaced traditional safety and immunogenicity, and safety and efficacy trials, to such a large extent. We speculate that the wholesale shift towards experimental medicine strategies is a manifestation of a limited funding environment, which has forced developers to adopt more cost-effective approaches to vaccine testing. One major disadvantage to cost-effective experimental medicine approaches is that we do not know if prevention of infection will result in prevention of disease. Reciprocally, it is possible that prevention of disease could be achieved with a vaccine that had no impact on infection and POI trials would triage out such candidates. Success of a vaccine candidate in a POI or POR trial would likely accelerate clinical development, but should failure in an experimental medicine trial halt progression in the traditional development pathway?

It also appears that the number and diversity of new TB vaccine candidates entering clinical trials has become increasingly limited, which might severely restrict the options to develop new TB vaccines aimed at a wide target spectrum of age (infants, adolescents, adults or the elderly), MTB exposure status (IGRA+, IGRA ^-^), and indication (POD, POR and therapeutic). No matter how promising an individual candidate may appear in pre-clinical studies, it would be high risk to commit resources only to development of a single candidate, which inevitably carries some risk of failure when safety, immunogenicity and efficacy are tested in humans. This consideration is perhaps most relevant to the highest priority of protecting previously BCG vaccinated, MTB-infected adults against progression to active TB disease.

Therefore, we propose that diversity is an essential quality of a healthy TB vaccine pipeline that will ultimately lead to a successful vaccine, or vaccines, that meet the needs of a variety of susceptible populations, including adults, children and HIV-infected persons living in TB endemic countries. If we accept this premise, it follows that we must accept the inevitable possibility of failure of individual candidates, or specific trial designs, to meet acceptable standards when tested for safety, immunogenicity or efficacy in human populations. We want to accept risk, after serious evaluation of all issues including biological, medical, ethical and legal aspects. We need not fear failure, since progression in our field depends on our willingness to evaluate and accept risk, provided that we learn from each clinical trial, and collect and store sufficient data and samples to improve future chances of success. Perhaps the most illustrative example of ‘failing well’ is the infant trial of the MVA85A candidate, which conclusively failed to demonstrate added protection to that provided by newborn BCG vaccination, but taught invaluable lessons about conduct of infant vaccine trials ^42, 47– 49^, pre-clinical animal models ^28^, community engagement ^47^, endpoint determination ^50, 51^, and biomarkers of risk for TB ^22, 52^.

We propose that logical application of Stage Gate criteria to de-risk and progress a number of diverse vaccine candidates through human trials, in the knowledge that most will fail, is a necessary and efficient strategy to achieve the ultimate goal of a successful TB vaccine. This approach does carry the responsibility, not only to learn iteratively from past failures, but to be prepared to adapt rapidly and decisively in the face of new data. We advocate for empiric advancement of promising candidates into clinical trials, in parallel with iterative studies to better understand risk for and protection against TB. Conversely, we propose that application of our field’s limited global resources to development of only a small number of very carefully selected candidates, no matter how promising, would lead to a shrinking, less diverse pipeline, increasingly vulnerable to the consequences of failure. In that context, excessive risk-aversion is itself a high-risk strategy, given the urgency for a new vaccine to impact on global burden of disease.

## Conclusions

This paper reviewed progress made in TB vaccine development since the initial Blueprint for the field was published in 2012. Then and now, a TB vaccine is inevitable to put an end to TB. As for many other infectious diseases, a safe, efficacious and affordable vaccine is an essential part of the solution. Getting there will not be easy, and tremendous progress has been made during the last five years. The TB vaccine community now has at its disposal a broad portfolio of platform technologies, vaccine antigens and insight into immunological mechanisms that can be leveraged to expedite TB vaccine development. However, the global TB vaccine pipeline has progressed much less than desired in recent years, and we hope that the new tools and insights from technologies and discovery research will feed into a rich and diverse pipeline in the future. Animal models will be vital to swiftly advance novel vaccine candidates into clinical trials. While the predictive value of animal models can ultimately only be validated by human efficacy data, the evaluation of vaccine candidates in a combination of rationally selected animal models provides an early gauge and Stage Gate. Similarly helpful in moving vaccines through the development path are biomarkers and immune correlates. Biomarker technology has matured enormously over the last five years, yielding tests that are ready to support pre-clinical and clinical development. Further validation of promising biomarkers could come from experimental medicine studies (including a human mycobacterial challenge model) and from larger clinical trials with an efficacy endpoint. Continued clinical research as well as advanced large scale field studies will be critical to validate animal models and biomarkers, establish proof of scientific concepts, and will ultimately yield an efficacy signal that the entire field is waiting for. We cannot afford to relent in this effort and must be willing to invest wisely, knowing that many trials will fail. Acceptance of risk and failure is an integral part of developing vaccines and the potential global public health impact of an effective vaccine should encourage us to continue to invest our intellectual and financial capital in TB vaccine development.

## Data availability

*All data underlying the results are available as part of the article and no additional source data are required.*
