# Supplementary material for: Progress and challenges in TB vaccine development
Source: F1000Res. 2018 Feb 16;7:199. [Version 1] doi: 10.12688/f1000research.13588.1 (PMC5850090; doi:10.12688/f1000research.13588.1)
Supplement: Peer review history

# Peer review: Referee response for version 1

**DOI:** 10.5256/f1000research.14760.r30908

**Published:** 2018-03-13

**Authors:** Ashley Birkett

**Affiliations:** 1 PATH’s Malaria Vaccine Initiative (MVI), Seattle, WA, USA

In this manuscript, the authors rationally assert that compelling biological evidence for the induction of protective immunity associated with BCG vaccination, as well as by natural exposure, strongly support the feasibility for development of an improved TB vaccine. The manuscript highlights progress since the 2012 publication of the Blueprint for TB Vaccine Development, both in TB-specific and non-specific areas, that are critical to inform the design and testing of novel vaccines approaches. Progress is highlighted in areas such as the understanding of the basis for protective immunity, vaccine design (i.e. antigens, platforms and adjuvants), and improved sophistication of clinical trial design (i.e. enhanced inclusion/exclusion criteria associated with specific endpoints) to reduce sample size and trial duration, and therefore costs.

 Despite the documented advances, the main theme of the paper is one of frustration with the slow pace in advancing existing candidates and in the repopulation of the global vaccine portfolio with vaccines built on diverse approaches that leverage new scientific learnings. The authors argue that risk aversion on behalf of funding agencies is a critical factor in this regard. While I cannot speak to the current funding environment for TB vaccine development (convincing data on reduced investments in this area was not provided), the authors, quite rightly, suggest that a step-wise advancement (requiring investment risk), is more likely than the sudden transformational breakthrough likely to align with a risk averse investment approach. Appropriately, the authors acknowledge the need to identify approaches that induce clinical efficacy (including via nascent human challenge models) to ‘back-validate’ non-clinical models and thereby improve their predictive value of clinical outcomes going forward.

 The manuscript would have benefited from a clearer communication on the Preferred Product Characteristics (PPCs) that underpin a (presumed) positive value proposition for the next generation target vaccines targeting the expressed indications, of which there were several.

I have read this submission. I believe that I have an appropriate level of expertise to confirm that it is of an acceptable scientific standard.

# Peer review: Referee response for version 1

**DOI:** 10.5256/f1000research.14760.r31832

**Published:** 2018-03-12

**Authors:** Rhea Coler

**Affiliations:** 1 Infectious Disease Research Institute (IDRI), Seattle, WA, USA; 2 Department of Global Health, University of Washington, Seattle, WA, USA; 3 PAI Life Sciences, Seattle, WA, USA

I reviewed the manuscript “Progress and challenges in TB vaccine development” and it seems to me that the objective of this review is well defined and clearly stated.

 The abstract is written clearly and objectively. Introduction is clear and presents a solid foundation for the review's objectives. The presentation of the various sub-topics is clear and the review of the bibliography is mostly complete and includes the main articles published in the area.

 The section on animal models could be elaborated to discuss in greater details mouse and guinea pig TB models, in addition to the authors’ thoughtful perspective on the use of NHPs.

 Some discussion of the intricate network of events modulating inflammation in the context of TB and how a better understanding of this will aid in the development of more effective vaccines and host-directed therapies to curb TB is warranted.

 The review should also be updated given the recent report of the first efficacy trial evaluating prevention of infection results for BCG revaccination or H4:IC31.

 Thank you for the opportunity to review this excellent work.

I have read this submission. I believe that I have an appropriate level of expertise to confirm that it is of an acceptable scientific standard.
